# ArchaeaHQ: a curated reference database of archaeal genomes

**DOI:** 10.1099/mgen.0.001884

**Published:** 2026-11-12

**Authors:** Dmitry Bespiatykh, Pedro Leão

**Affiliations:** 1 Department of Microbiology, Radboud Institute for Biological and Environmental Sciences, Radboud University, Nijmegen, Netherlands

**Keywords:** archaea, archaeal genomics, database, metagenomics

## Abstract

Archaea have proven to be major players in biogeochemical cycles across diverse ecosystems, yet we still see an underrepresentation of archaeal genomes in the datasets used by popular computational biology tools. Here, we present ArchaeaHQ, a quality-controlled, systematically curated reference database of 25,584 archaeal genomes compiled initially from 43,045 assemblies from all four archaeal kingdoms retrieved from NCBI: Methanobacteriati (Euryarchaeota), Thermoproteati (TACK), Nanobdellati (DPANN) and Promethearchaeati (Asgard). All genomes in the database passed standardized quality control, requiring ≥70% completeness and ≤10% contamination. A total of 44.0% of genomes in ArchaeaHQ achieved ≥90% completeness, while 92.7% exhibited ≤5% contamination. ArchaeaHQ comprises 18,701 metagenome-assembled genomes (MAGs; 73.1%) and 6,883 isolate genomes (26.9%). Approximately 76% of MAGs are assigned to 17 ecologically meaningful categories based on sampling origin, and around 65% of genomes include geographic metadata. ArchaeaHQ is available at https://doi.org/10.6084/m9.figshare.32266599 for download and at https://vee-lab.eu/archaeahq for online browsing. This resource was designed to provide an analysis-ready reference set for metagenomic, biogeochemical and ecological studies, comparative genomics analyses and the development of archaeal-specific bioinformatic tools.

**Impact Statement**

Archaea are key drivers of the global carbon, nitrogen and sulphur cycling, including major processes such as methane production and oxidation, yet their genomes remain underrepresented and inconsistently curated in the public databases that power modern computational biology research. We present ArchaeaHQ, a quality-controlled, systematically curated reference set of 25,584 archaeal genomes spanning all four archaeal kingdoms, each passing standardized completeness and contamination thresholds and enriched with environmental and geographic metadata. By providing an analysis-ready, downloadable resource compatible with standard pipelines, ArchaeaHQ fills the gap between taxonomy-focused frameworks and unfiltered genome archives, supporting metagenomic classification, biogeochemical and ecological studies, comparative genomics and the development of archaeal-specific bioinformatic tools.

## Data Summary

ArchaeaHQ is available at https://doi.org/10.6084/m9.figshare.32266599. All genome assemblies are publicly available through NCBI GenBank under their original accession numbers. The custom scripts used for metadata extraction, environmental classification and geographic mapping are available at https://github.com/Pedrolleao/genome-metadata-toolkit. A browsable version of the ArchaeaHQ is also available through our laboratory’s website at https://vee-lab.eu/archaeahq. A script to direct update the database is available in the GitHub repository (https://github.com/Pedrolleao/archaeahq-update)

## Introduction

From the production of methane in water bodies to the oxidation of ammonia in the ocean, Archaea drive biogeochemical processes that have been shaping life on Earth from its inception to modern days. Today, methanogenic archaea are responsible for the production of approximately 1 Gt of methane per year [1]. In contrast, anaerobic methane-oxidizing archaeaform syntrophic associations with sulphate-reducing bacteria at the sediment–water interface. These consortia consume methane ascending from marine sediments and thereby act as a natural filter that limits methane emissions [2, 3]. Archaea within the kingdom Methanobacteriati (Euryarchaeota) play a key role in the global carbon cycle through methane production and oxidation. Ammonia-oxidizing archaea are representatives from the kingdom Thermoproteati (TACK), known for catalysing the conversion of ammonia to nitrite, a key step in nitrification, and thereby coupling carbon and nitrogen cycles at global scales [4, 5].

In the last 10 years, the metagenomic revolution revealed that the cultivated fraction of archaeal diversity represents a minor, phylogenetically skewed, sample of what exists in nature [6, 7]. Among other discoveries, metagenomics introduced us to two new archaeal kingdoms: Nanobdellati (DPANN) and Promethearchaeati (Asgard). DPANN archaea are ultra-small archaea with extremely reduced genomes that appear to live in synergy (as episymbionts or parasites) with other archaeal groups [8, 9]. Asgard archaea represent the closest known archaeal relatives of eukaryotes [10, 11], and their study has continued to refine eukaryogenesis models bringing us closer to uncovering the origin of complex life on Earth [10, 12, 13].

The impact of this revolution is reflected in online public genome resources. As of September 2026, NCBI GenBank hosted 43,045 archaeal genome assemblies, with more than 70% being metagenome-assembled genomes (MAGs) recovered from environmental samples rather than cultivated isolates. Yet the addition of assemblies into public archives proceeds without systematic quality filtering, metadata standardization or redundancy control, meaning that raw public archives are simultaneously a valuable scientific resource and a risky analytical mixture of high-quality genomes, fragmented or contaminated assemblies and redundant sequences with inconsistent or absent environmental data. Several genomic resources address some of these issues, but none was designed to serve as a dedicated, analysis-ready high-quality archaeal genome reference set.

NCBI GenBank [14] provides the most comprehensive assembly archive, but quality, completeness and associated metadata vary enormously across deposited records, and no curated archaeal genome subset is available.

The Genome Taxonomy Database (GTDB) [15] is an ongoing census of bacterial and archaeal diversity through a phylogenetically consistent, rank-normalized, genome-based taxonomy. It offers archaeal genomes of selective taxonomic representatives under a reclassified, phylogenetically coherent taxonomy. This is an extremely valuable resource, but GTDB serves primarily as a taxonomy framework reference rather than a downloadable, quality-filtered collection of archaeal genomes linked to environmental data. Besides, GTDB’s reclassified taxonomy diverges systematically from the latest NCBI taxonomy, creating incompatibilities with the NCBI accessions, BioSample metadata and taxon identifiers used by many widely deployed bioinformatic tools.

The Integrated Microbial Genomes and Microbiomes system (IMG/M) [16], maintained by the Joint Genome Institute (JGI), contains a large collection of archaeal MAGs with rich environmental context but is not publicly downloadable as a precompiled genome set with standardized metadata, limiting its utility to large-scale command-line-driven workflows. Collectively, these limitations highlight the need for a dedicated, quality-controlled archaeal genome resource with broad taxonomic and environmental representation that is directly downloadable and compatible with standard bioinformatic pipelines, thereby supporting reliable comparative and functional analyses across the archaeal domain.

Here, we introduce ArchaeaHQ, a quality-controlled, curated reference database of 25,584 archaeal genomes compiled from 43,045 assemblies retrieved from NCBI, containing all four archaeal kingdoms: Methanobacteriati (Euryarchaeota), Thermoproteati (TACK), Nanobdellati (DPANN) and Promethearchaeati (Asgard). ArchaeaHQ is designed for direct use in metagenomic classification, biogeochemical and ecological context studies, comparative genomics and the development of archaeal-specific computational tools.

## Results and discussion

### ArchaeaHQ: a high-quality archaeal genome database

Publicly available genome repositories such as NCBI GenBank are expanding rapidly in the number of archaeal assemblies deposited, but the quality, completeness and associated environmental metadata of these assemblies vary enormously. No dedicated, systematically curated archaeal genome reference set has previously been available to support environmental and ecological research applications. The initial step of the ArchaeaHQ development was the recovery of archaeal genome assemblies deposited in NCBI as of September 2026, followed by a multi-step curation pipeline (Fig. 1a) that applied standardized quality thresholds to remove species redundancy, low-quality assemblies and retain genome representatives of the broad taxonomic and environmental spectrum of the known archaeal kingdoms.

**Fig. 1.**
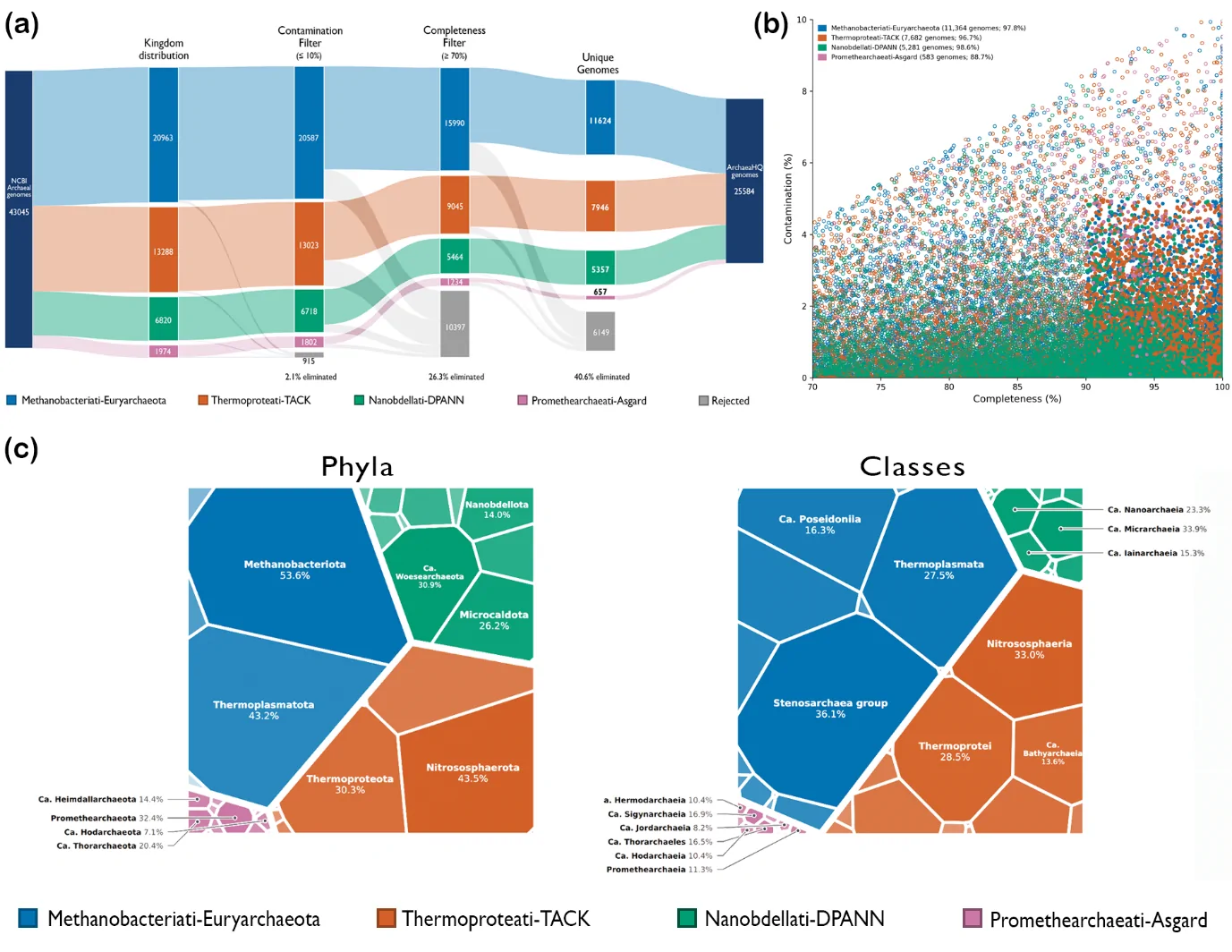
ArchaeaHQ captured a high-quality archaeal genome set after systematic curation removed nearly 40% of NCBI archaeal assemblies. (**a**) Sankey diagram showing the filtering and curation workflow used to select genomes included in ArchaeaHQ. (**b**) Completeness and contamination distribution of the curated ArchaeaHQ genomes using the Parks genome quality score (completeness – 5 × contamination) [15]. (**c**) Phyla and class proportional distribution of the genomes in ArchaeaHQ. The names and corresponding percentage of the taxons that compose 70% of each archaeal kingdom are present in the figure. Figures at the order and family organization level are available in Fig. S1, available in the online Supplementary Material. Bars in (**a) **and points in (b) are colour-coded by archaeal kingdom: blue, Methanobacteriati-Euryarchaeota; orange, Thermoproteati-TACK; pink, Promethearchaeati-Asgard; green, Nanobdellati-DPANN. The final curated metadata for all 25,584 archaeal genomes included in ArchaeaHQ are provided in Table S1.

The final database comprised 25,584 genomes, a 40.6% reduction from the 43,045 assemblies initially recovered from NCBI. The database includes both cultivated isolate genomes (*n*=6,883; 26.9%) and MAGs (*n*=18,701; 73.1%), highlighting the fact that the vast majority of archaeal diversity remains uncultivated and must be accessed through culture-independent approaches.

The initial NCBI dataset contained assemblies from 33,318 unique species-level taxa from all 4 archaeal kingdoms, confirming that the raw archive captures substantial archaeal species diversity. After excluding identical genomes, ArchaeaHQ comprised the following high-quality, unique genome assemblies: Euryarchaeota (*n*=11,624), TACK (*n*=7,946), DPANN (*n*=5,357) and Asgard (*n*=657).

### Genome quality metrics

The quality metrics of the retained genomes in ArchaeaHQ reflect the stringency of the curation pipeline (Fig. 1a) and provide a robust foundation for downstream analyses that require reliable assembly completeness and low levels of sequence contamination. Completeness across the 25,584 genomes ranges from 70% (the minimum threshold) to 100%, with a mean of 87.5% and a median of 88.3%. A total of 11,259 genomes (44.0%) reach ≥90% completeness, meeting the completeness sub-criterion of the Minimum Information about a Metagenome-Assembled Genome (MIMAG) high-quality standard [17]. Contamination is correspondingly low: values range from 0 to 10% (the maximum threshold), with a mean of 1.54% and a median of 0.78%, and 23,724 genomes (92.7%) exhibit ≤5% contamination. A total of 97.8% of Euryarchaeota, 96.7% of TACK, 98.6% of DPANN and 88.7% of Asgard genomes present in ArchaeaHQ have a Parks genome quality score (completeness – 5 × contamination) [15] above 50 and are shown in Fig. 1(b).

Complementing these assembly-quality metrics, taxonomic profiling revealed that ArchaeaHQ spans 4 kingdoms, 39 phyla, 51 classes, 84 orders, 119 families and 307 genera (Table S1). However, the proportion of genomes assigned to a named taxon declined from 97.4% at the phylum level to 24.9% at the genus level, highlighting the limited resolution of NCBI taxonomic annotations for many genomes in the database.

### Environmental diversity captured in ArchaeaHQ

The in-depth curation of the environmental context of the genomes is what sets ArchaeaHQ apart from other archaeal genome resources, making it directly applicable to ecological and biogeochemical research (Table S1). The predominance of MAGs in the dataset (73.1%) reflects the current state of archaeal genomics, where cultivation-independent sequencing has been the primary driver of novelty in the archaeal community, particularly for lineages belonging to the DPANN and Asgard kingdoms that have few cultured representatives that require extensive time and resources for their cultivation [13, 18]. The inclusion of both MAGs and isolate-derived genomes in ArchaeaHQ ensures that the database captures both the genomic robustness present of isolate-derived assemblies and the ecological context provided by metagenomic resources.

The environmental metadata provided by ArchaeaHQ (Table S2) reveals strong representation of extreme and geochemically active environments. Terrestrial hot springs represent the largest environmental source (*n*=4,376 genomes), followed by terrestrial soil (*n*=2,339), marine water column (*n*=2,915) and deep-sea hydrothermal vent (*n*=2,231) (Fig. 2). The dominance of geochemically extreme habitats is consistent with the historical report of high relative abundance of archaeal lineages in these extreme environments, making the recovery of these MAGs less complex than in environments where the bacterial population dominance is extremely high [19, 20]. The large number and broad diversity of high-quality archaeal genomes highlight the value of ArchaeaHQ for studying archaeal contributions to global biogeochemical cycling. TACK archaea are particularly well represented in terrestrial hot spring (36.55%), terrestrial soil (13.55%) and hydrothermal vent (11.11%) assemblies, while Euryarchaeota can be found in a broad array of environments; most of its assemblies are originated from the marine water column (17.98%), animal-host-associated samples (10.40%) and terrestrial soil (8.71%). DPANN genomes are mainly recovered from subsurface environments (24.57%), acid mine drainage (15.42%) and terrestrial hot springs (14.84%), while Asgard genomes are predominantly recovered from mangroves (31.66%), marine sediment (28.46%) and cold seep systems (10.35%) (Table S2).

**Fig. 2.**
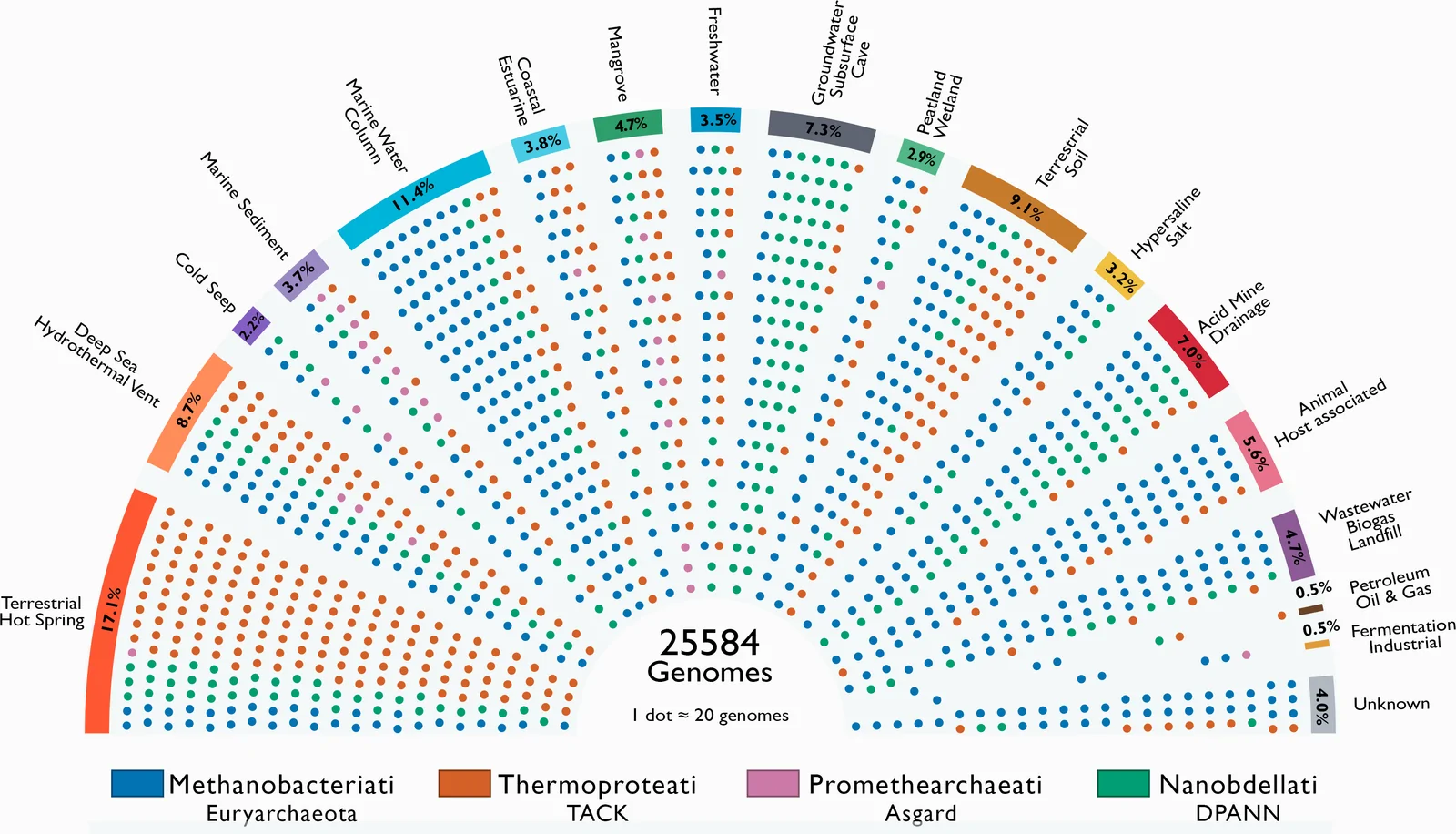
ArchaeaHQ genomes are strongly represented by hot springs, soils, marine waters and hydrothermal vents. The values on the bars below each environment represent the percentage of all archaeal genomes within that environmental category. Points under the area covering each environment represent roughly 20 genomes of the corresponding archaeal kingdom and are colour-coded by archaeal kingdom: blue, Methanobacteriati-Euryarchaeota; orange, Thermoproteati-TACK; pink, Promethearchaeati-Asgard; green, Nanobdellati-DPANN. The raw data used to produce this figure can be found in Table S3.

Beyond genomes recovered from extreme environments, ArchaeaHQ also contains several genomes recovered from soil (*n*=2,339), marine (*n*=2915 from water column; *n*=945 from marine sediment), freshwater (*n*=886) and coastal/estuarine (*n*=982) environments which provides a robust foundation to explore biogeochemical cycle dynamics. Archaea in these classic biomes play major roles in carbon, nitrogen and sulphur cycling. ArchaeaHQ leverages high-quality genomes of key biogeochemical players to enable quantitative genomic analyses, metabolic pathway reconstruction and a deeper understanding of archaeal functional evolution.

Geographic metadata are available for 16,617 genomes (65% of the database), enabling spatial analyses of archaeal biogeography and facilitating the integration of ArchaeaHQ genomes with environmental contextual data in macroecological and comparative genomic studies (Fig. 3). BioSample-level geographic metadata were verified and retained from NCBI records, providing a practical advantage over unfiltered NCBI downloads, in which geographic metadata are inconsistently available. To accompany the environmental metadata, 21,972 16S rRNA gene sequences were recovered from 17,255 genomes present in ArchaeaHQ. Information about the size of these sequences is available in Table S5. The 16S rRNA sequences are included in the ArchaeaHQ dataset and are also available on Figshare (Archaea_HQ-16S.fasta; https://doi.org/10.6084/m9.figshare.32266599).

**Fig. 3.**
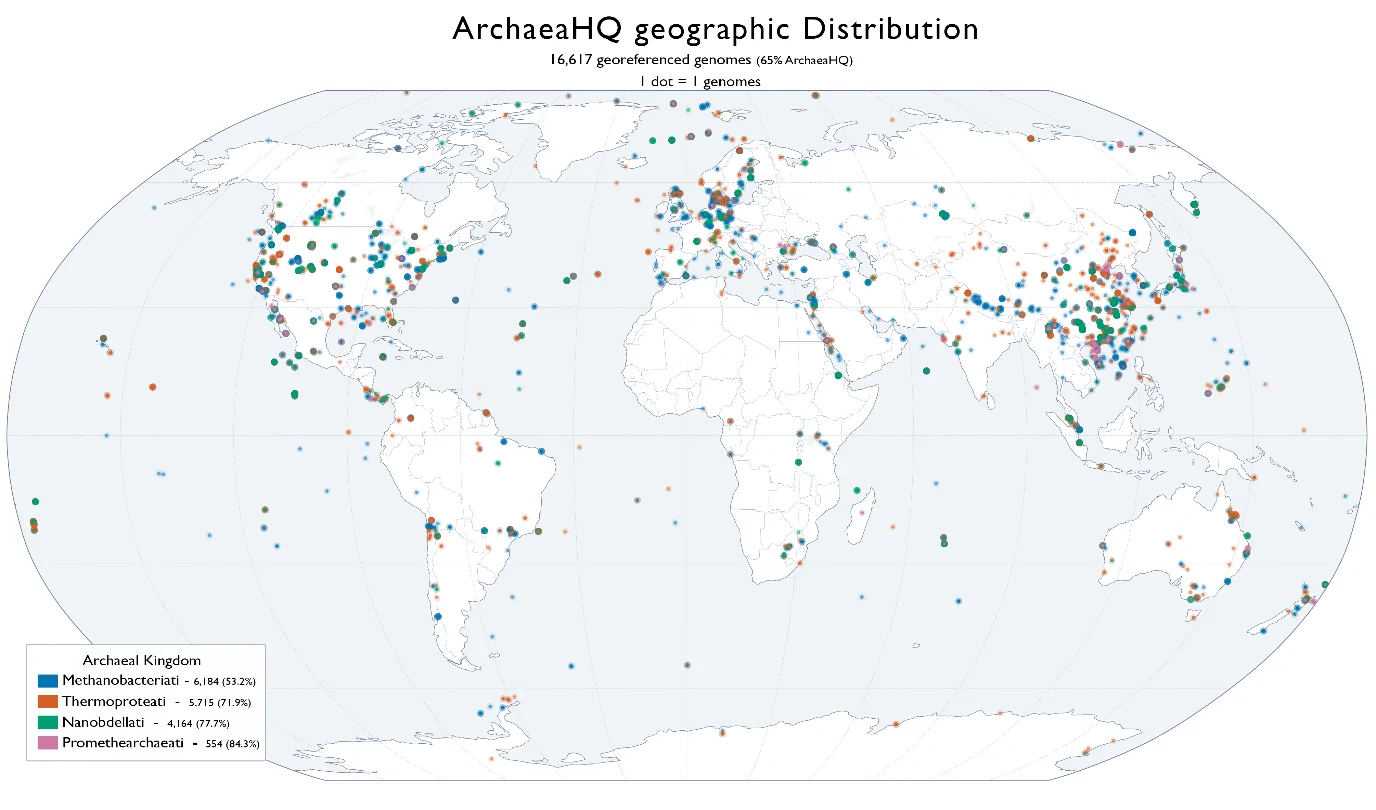
Geographic distribution of the sampling location associated with 16,617 of archaeal genomes present in ArchaeaHQ (65%). Each dot represents one genome, with regions where multiple genomes were isolated presenting a growing halo proportional to the amount of genomes isolated from that coordinate. Points are colour-coded by archaeal kingdom: blue, Methanobacteriati-Euryarchaeota; orange, Thermoproteati-TACK; pink, Promethearchaeati-Asgard; green, Nanobdellati-DPANN. All the geographic information used in this graph can be found in Table S1.

Altogether, the global environmental diversity combined with curated quality control and taxonomic information positions ArchaeaHQ as a valuable resource for archaeal genomics, with direct applications in metagenomic classification, biogeochemical functional gene surveys, comparative functional annotation and the development of archaeal-specific bioinformatics tools, including machine learning models for gene prediction and functional annotation.

### Relationship with existing archaeal genomic resources

Existing archaeal genome resources provide valuable but incomplete solutions for large-scale comparative and functional analyses. GTDB (Release 09-RS220) offers a phylogenetically consistent framework with representative genomes but is primarily designed for taxonomy rather than as a downloadable, analysis-ready dataset and does not prioritize standardized environmental information or compatibility with NCBI-based workflows. The IMG/M database contains extensive archaeal genome collections with rich contextual data yet lacks a unified, quality-filtered and easily accessible reference set suitable for direct use in scalable bioinformatic pipelines.

ArchaeaHQ addresses these limitations by providing a curated, quality-controlled and fully downloadable archaeal genome database ready to be used. It maintains compatibility with NCBI taxonomy and publicly available accessions (e.g. BioProject and BioSample) (Table S1), applies consistent quality thresholds while preserving diversity from underrepresented lineages such as DPANN and Asgard groups and integrates environmental information as a core feature. In addition, users can update ArchaeaHQ by retrieving newly available archaeal genomes from NCBI and adding or replacing genomes in the database using the same robust quality control and metadata retrieval procedures applied in this study (see Methods). The database update script and a step-by-step guide are available in the GitHub repository (https://github.com/Pedrolleao/archaeahq-update). By combining accessibility and standardization, ArchaeaHQ fills a critical gap between taxonomy-focused resources and fragmented genome repositories, enabling a reliable method for the incorporation of archaea in future comparative genomics studies and the development of computational biology tools.

## Methods

### Genome retrieval and dataset compilation

All archaeal genome assemblies deposited in NCBI GenBank were retrieved in September 2026 using the NCBI Datasets command-line interface (v.15.19.1) [21] with the ‘datasets download genome taxon’ command. Assemblies were downloaded independently for each of the four recognized archaeal kingdoms: Methanobacteriati (Euryarchaeota; taxon ID 3366610), Thermoproteati (TACK; taxon ID 1783275), Nanobdellati (DPANN; taxon ID 1783276) and Promethearchaeati (Asgard; taxon ID 1935183), yielding a combined dataset of 43,045 genome assemblies. Genome sequences (FASTA format) and associated BioSample metadata, including NCBI assembly accession, BioProject, BioSample identifier, NCBI taxonomic assignment, isolation source, metagenome source label, broad and local environment descriptors (ENVO terms) and geographic coordinates (geo_loc_name, lat/lon), were extracted for each assembly using custom scripts from the genome-metadata-toolkit (https://github.com/Pedrolleao/genome-metadata-toolkit).

### Genome quality assessment

Genome completeness and contamination were estimated for all assemblies using CheckM2 v1.0.1 [22] with default parameters and using the general archaeal marker gene set. Assemblies were retained for downstream analysis only if they simultaneously presented both completeness ≥70% and contamination ≤10%. These thresholds represent a permissive but meaningful quality gate that captures the range of archaeal genomic diversity while excluding highly fragmented or heavily contaminated assemblies. The 70% completeness minimum sits between the high- and medium-quality thresholds of the MIMAG standard [17], while contamination ≤10% is more permissive, allowing us to capture a broad diversity of archaeal genomes while eliminating heavily contaminated assemblies.

NCBI contamination warnings are reported in Table S1, but they were not used as an exclusion criterion during the curation of ArchaeaHQ, as genome quality was independently assessed using CheckM2 and ANI analyses rather than NCBI’s Foreign Contamination Screen (FCS).

### Redundancy removal and representative genome selection

Inter-genome comparisons and species-level clustering were performed using dRep v3.6.2 [23]. Primary clustering was performed using Mash distance sketches; within each primary cluster, pairwise average nucleotide identity (ANI) and reciprocal alignment coverage were computed using NUCmer v.4.0.1 [24]. Two tiers of genomic redundancy were resolved using a Union-Find (disjoint-set) algorithm to maintain consistent clustering relationships:

1. Identity-level redundancy (duplicate submissions): genome pairs with ANI ≥0.999 and reciprocal alignment coverage ≥0.99 were grouped as identical. Because RefSeq genome assemblies (GCF_ prefix) are derived from and explicitly paired by NCBI with their corresponding GenBank assemblies (GCA_ prefix), GCA/GCF pairs sharing the same numeric assembly identifier were treated as alternative database representations of the same underlying assembly [25]. Although paired GenBank and RefSeq assemblies are generally sequence-identical, NCBI curation can occasionally introduce sequence-level differences between them, for example, through removal of contaminating sequence. Consequently, such paired accessions were grouped at the identity-redundancy stage regardless of measured reciprocal alignment coverage. When selecting the preferred representative from a paired GCA/GCF group, the most recent RefSeq version was retained in the absence of a dRep-designated representative.
2. Species-level redundancy: genome pairs with ANI≥0.95 and reciprocal alignment coverage ≥0.95 were assigned to the same species-level cluster, consistent with the widely applied prokaryotic species boundary [26]. Within each species-level cluster, the genome selected by dRep as representative based on a scored composite metric incorporating completeness, contamination and assembly contiguity (contig N50) was retained in ArchaeaHQ. For species clusters lacking a dRep representative (e.g. due to all cluster members being outcompeted by a genome of higher quality in the ANI comparisons), the most recently curated RefSeq version within the cluster was retained. Genomes not grouped with any other assembly during Mash-distance primary clustering (singletons) were retained directly in ArchaeaHQ as unique representatives.

The genomes retained as part of ArchaeaHQ are analysed using barrnap/ARAGORN [27] for rRNA/tRNA annotation and sequence recovery.

### Geographic metadata curation

Geographic coordinates were extracted directly from the lat\_lon field of NCBI BioSample records associated with each retained assembly. Records were flagged as ‘georeferenced’ only when a valid, parseable decimal degree coordinate pair was present; entries containing placeholder values (e.g. ‘0 N 0 E’, ‘missing’ or equivalent uninformative strings) were excluded. Geographic coverage was computed per archaeal kingdom to characterize differential metadata availability across the archaeal domain.

### User-driven database update

For users to keep the database updated with newly deposited archaeal assemblies on NCBI, we developed the archaeahq-update tool (https://github.com/Pedrolleao/archaeahq-update). This tool identifies archaeal genome assemblies deposited at NCBI since the previous database version and evaluates them under the same criteria used to build ArchaeaHQ v1.0. Briefly, candidate assemblies were downloaded via the NCBI Datasets CLI [21] and screened through a quality gate (CheckM2 [22]; completeness ≥70%, contamination ≤10%), a redundancy filter against both the existing database and other genomes in the same batch (skani [28]; ≥99.9% ANI and ≥99% aligned fraction defined as identical, ≥95%/≥95% as same species), rRNA/tRNA annotation (barrnap/ARAGORN) [27] and environmental classification using the same rule-based classifier as the original build. Genomes passing all filters are flagged for addition (add), and newer assembly versions of existing genomes in the database that passed the quality gate are flagged as replacements (replace). The addition of all genomes flagged as ‘add’ or ‘replace’ can be accomplished with one command, ‘*archaeahq-update release*’, to generate a new set of amino acid (faa), nucleotide (fna) and 16S rRNA (Archaea_HQ-16S.fasta) sequences of the genomes in the updated version of ArchaeaHQ and an updated version of Table S1 (Archaea_HQ-16S.tsv).

## Supplementary material

10.1099/mgen.0.001884 Fig. S1.

10.1099/mgen.0.001884 Supplementary Material 1.

## Abbreviations

ANI: average nucleotide identity
GTDB: Genome Taxonomy Database
IMG/M: Integrated Microbial Genomes and Microbiomes system
MAGs: metagenome-assembled genomes
MIMAG: Minimum Information about a Metagenome-Assembled Genome
