# Supplementary material for: ArchaeaHQ: a curated reference database of archaeal genomes
Source: Microb Genom. 2026 Nov 12;12(11):001884. doi: 10.1099/mgen.0.001884 (PMC13758933; doi:10.1099/mgen.0.001884)
Supplement: Supplementary file 1 [file mgen-12-01884-s001.pdf]

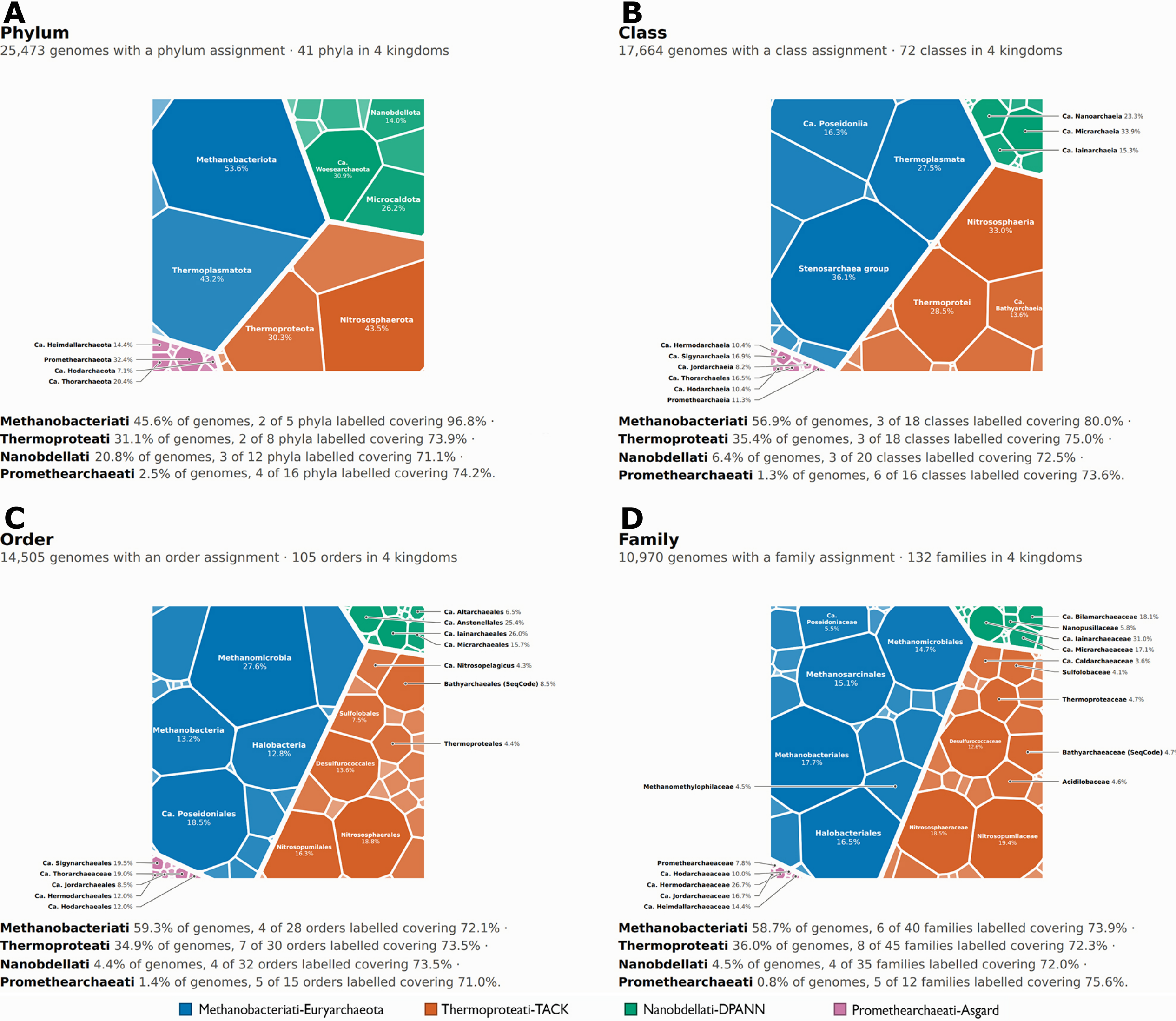

Supplementary Figure 1: Taxonomic diversity and distribution of genomes in ArchaeaHQ. The area of each tile is proportional to the relative abundance of genomes assigned to that taxon within the database. Hierarchical treemaps illustrating the phylogenetic distribution at the (a) Phylum, (b) Class, (c) Order, and (d) Family levels, encompassing 4 archaeal kingdoms (Methanobacteriati - Euryarchaeota in Blue; Thermoproteteati - TACK in Orange; Nanobdellati-DPANN in Green; Promethearchaeati - Asgard in Pink). Total assigned genomes and taxonomic counts are indicated at the top of each panel. Statistics below each panel denote the percentage of total genomes contributed by major constituent lineages, the number of labelled taxa, and their cumulative percentage coverage.
